# Synchrotron X-ray multi-projection imaging (XMPI) for high-resolution 4D characterization of multiphase flows

**DOI:** 10.1007/s00348-026-04271-6

**Published:** 2026-07-23

**Authors:** Tomas Rosén, Zisheng Yao, Jonas Tejbo, Patrick Wegele, Julia K. Rogalinski, Frida Nilsson, Kannara Mom, Zhe Hu, Samuel A. McDonald, Kim Nygård, Andrea Mazzolari, Alexander Groetsch, Korneliya Gordeyeva, L. Daniel Söderberg, Fredrik Lundell, Lisa Prahl Wittberg, Eleni Myrto Asimakopoulou, Pablo Villanueva-Perez

**Affiliations:** 1 Department of Fibre and Polymer Technology, KTH Royal Institute of Technology, Stockholm, Sweden; 2 Synchrotron Radiation Research and NanoLund, Lund University, Lund, Sweden; 3 Department of Engineering Mechanics, KTH Royal Institute of Technology, Stockholm, Sweden; 4 TIMC, Université Grenoble Alpes, Grenoble, France; 5 MAX IV Laboratory, Lund University, Lund, Sweden; 6 Department of Physics and Earth Science, Ferrara University, Ferrara, Italy

## Abstract

Multiphase flows where particles, bubbles, or droplets are suspended in a fluid govern critical processes in biology, medicine, materials processing, and geophysics. However, observing their microscale dynamics in opaque systems has remained a fundamental challenge. We present Synchrotron X-ray Multi-Projection Imaging (XMPI), a novel approach enabling four-dimensional (3D + time) tracking of microparticles in visibly opaque suspension flows without requiring sample rotation. By capturing simultaneous projections from multiple angles using beam-split X-rays at synchrotron facilities, we resolve instantaneous particle positions and trajectories in opaque fluids such as blood. We demonstrate the potential of XMPI through individual particle tracking velocimetry (3D PTV) in dilute conditions, as well as multi-projection image velocimetry in dense suspensions. The methodology provides otherwise inaccessible experimental validation for particle-resolved computational fluid dynamics models and allows, e.g., observation of inertial focusing effects and microstructural dynamics relevant to suspension rheology and biomedical flows. This work paves the way for high-resolution, time-resolved 4D imaging of complex multiphase flows across a range of scientific and industrial applications. Combining XMPI with recent AI-supported 4D reconstruction algorithms opens a new spatiotemporal frontier for high-speed, rotation-free microtomography.

**Graphical abstract:**

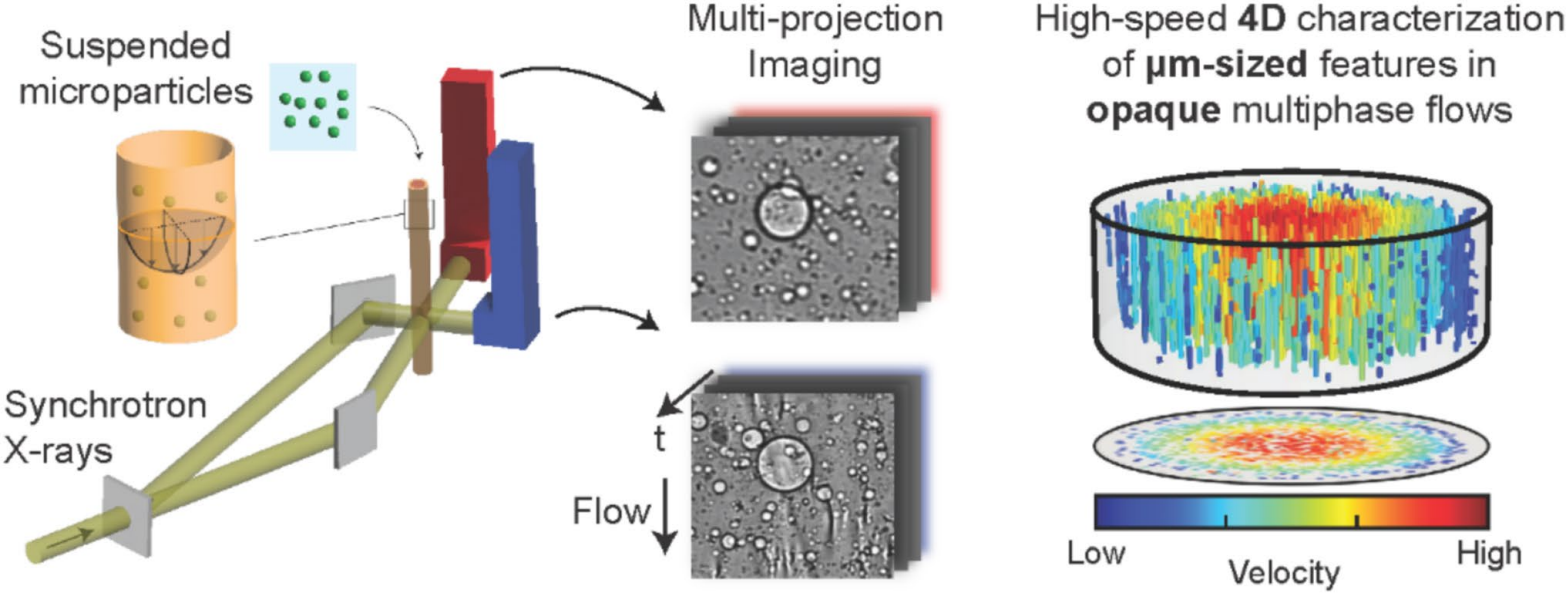

**Supplementary Information:**

The online version contains supplementary material available at 10.1007/s00348-026-04271-6.

## Introduction

Multiphase flows—involving particles, bubbles, or droplets suspended within a fluid—govern critical processes across biology, industry, and geophysics (Powell 2008; Poelma 2020). Examples span an exceptionally wide range of materials: blood, ink, paint, paper pulp, mud, lava, concrete, slurries, ketchup, and toothpaste all represent dense suspensions where microscopic particles govern macroscopic behavior. Although these flows appear continuous at macroscopic scales, they consist of interacting micrometer-sized components whose local dynamics strongly affect the bulk properties of the system. These microscale interactions influence processes such as clot formation in blood, landslides in mud, fiber alignment in papermaking, and flow resistance in dense industrial suspensions (Stickel and Powell 2005; Butler and Snook 2018; Lundell et al. 2011; Ness et al. 2022).

Understanding the physical behavior of these complex systems requires capturing their full four-dimensional (3D + time) dynamics at micrometer resolution. The ability to resolve how particles move, collide, and arrange within dense suspensions provides critical insight for predicting effective viscosity, flow-induced microstructures, and transitions to solid-like states such as jamming or aggregation. Despite advances in theoretical modeling and computational simulations (Freund 2014; Aidun and Clausen 2010; Sommerfeld 2017), experimental measurements remain essential for validation and for providing ground truth for these models.

Obtaining such time-resolved 3D experimental information for dense suspensions remains technically demanding (Powell 2008; Poelma 2020). Optical methods, such as laser-based particle tracking or optical coherence tomography (OCT), are limited to transparent or semi-transparent systems, often requiring index-matching strategies that are not applicable to many real-world suspensions (Cheng et al. 2011; Zade et al. 2018; Byron and Variano 2013; Haavisto et al. 2014). Other approaches, such as ultrasound Doppler velocimetry (UDV), magnetic resonance velocimetry (MRV), and electrical capacitance tomography (ECT), can access opaque systems but typically yield limited spatial or temporal resolution and often only provide averaged or indirect flow information (Poelma 2017; Fukushima 1999; Ma et al. 2019; Markl et al. 2011; Van Ooij et al. 2012; Zhang et al. 2014).

X-ray imaging offers a fundamentally different approach to overcome the optical opacity of these systems. Owing to their short wavelength and strong penetration capability, X-rays enable high-resolution imaging of opaque systems, providing access to multiphase flows at the micrometer scale (Heindel 2024; Kastengren and Powell 2014), including blood flows (Kim and Lee 2006; Lee et al. 2010; Irvine et al. 2008), cavitation flows (Jahangir et al. 2019), foams (Schott et al. 2023), drainage dynamics in porous media (Tekseth et al. 2024), and fluidized bed reactors (Mudde 2010). In addition, X-ray imaging is not affected by refractive distortions from curved or inclined channel walls, which commonly limit optical methods. This makes X-ray-based techniques particularly robust for studies involving complex geometries or non-planar interfaces. In these studies, X-rays were generated through lab devices like tube sources and electron guns, or advanced X-ray sources such as synchrotron light sources and X-ray free-electron lasers (XFELs). Yet, capturing fully time-resolved 4D information using X-rays has remained limited.

State-of-the-art 4D X-ray approaches acquire time-resolved X-ray images (radiographs) at multiple angles by either rotating the sample, i.e., computed tomographic X-ray imaging (XCT) (Withers et al. 2021), or by subjecting the sample to multiple beams from different directions (Heindel et al. 2008). Although time-resolved 4D XCT has been demonstrated for multiphase flows (Mäkiharju et al. 2022), the rotation limits its applicability to cases where the time scale for the flow is significantly longer than the time scale for rotation. The latter also poses a restriction since rapid rotation induces inertial forces in the rotating frame-of-reference that can lead to secondary flows. Alternatively, multiple X-ray beams can illuminate a stationary sample simultaneously and orthogonally, as demonstrated with laboratory sources (Chen et al. 2019; Bieberle and Barthel 2016; Neumann et al. 2019). For example, electron guns can generate an X-ray fan around the sample to provide a single 2D slice with kHz temporal resolution but millimeters spatial resolution (Bieberle and Barthel 2016; Neumann et al. 2019). Multiple state-of-the-art X-ray lab sources can then be mounted for stereographic particle tracking in 4D (Chen et al. 2019) to retrieve spatiotemporal resolutions on the order of 50 µm at 300 Hz (Zwanenburg et al. 2021; Vavřík et al. 2017). In short, such systems are suitable for tasks such as 3D particle tracking with a field-of-view of ~ 10 cm and a moderate spatiotemporal requirement, but the spatiotemporal resolution they can provide is ultimately limited by the flux from the lab-based X-ray source (Heindel 2011; Aliseda and Heindel 2021; Villafañe et al. 2025). XFELs and synchrotron light sources provide extremely high fluxes compared to lab-based X-ray sources that can enhance the spatiotemporal resolution to sub-micrometer resolution and kHz acquisition rates and beyond (Liang et al. 2023; Asimakopoulou et al. 2024; Villanueva-Perez et al. 2023; Olbinado and Rack 2019; Campbell et al. 2021). Despite these capabilities, multiphase flows at such facilities have so far been limited either to time-resolved 2D radiography of rapid fluid motion without sample rotation (Tolfts et al. 2024), or to 4D XCT of slow flow phenomena with slow sample rotation (Schott et al. 2025). The necessity of sample rotation to obtain 4D information has been preventing full exploitation of the high flux available at synchrotron and XFEL facilities. Consequently, the only applications that have thus far fully benefited from these high fluxes are highly deterministic and repeatable phenomena that can be reconstructed in 4D through repeated 2D radiography at different rotation angles (Tekseth et al. 2024). Thus, a rotation-less approach will open a new frontier of spatiotemporal resolution for multiphase flows by fully taking advantage of the capabilities of XFELs and synchrotron radiation facilities.

X-ray multi-projection imaging (XMPI) at synchrotron light sources and XFELs was recently demonstrated (Villanueva-Perez et al. 2018; Hoshino et al. 2011; Duarte et al. 2019), enabling 4D movies with micrometer resolution and kHz frame rates (and beyond) without rotating the sample (Liang et al. 2023; Asimakopoulou et al. 2024; Villanueva-Perez et al. 2023). This technique splits a single X-ray pulse to provide angularly separated beams, allowing single-shot 3D imaging and unparalleled 4D acquisition rates.

However, XMPI results in limited volumetric information due to the low number of projections. Thus, novel 4D reconstruction algorithms have been developed (Zhang et al. 2023, 2024) that combine ideas from the following techniques: (i) iterative 3D reconstruction (Herman 2009), like algebraic reconstruction, (ii) a combination of different experiments and samples (Punjani and Fleet 2023), such as cryo-electron microscopy, (iii) physical priors (Goodman 2005), i.e., considering the interaction and propagation of X-rays with matter, and (iv) deep-learning concepts to reconstruct 3D and 4D from sparse projections (Mildenhall et al. 2021), such as neural radiance fields. Combining these algorithms with XMPI opens new possibilities for studying time-resolved micron-sized features of dense particle suspension flows at kHz acquisition rates (and beyond) (Rogalinski et al. 2026; Witte et al. 2026). It thus paves the way to three-dimensional studies of high-speed and turbulent flows while eliminating experimental artifacts and limitations.

Here, we demonstrate how XMPI enables direct, rotation-free 4D particle tracking in both dilute and concentrated suspensions, including complex fluids such as human blood. We show that individual microparticles can be tracked in real time, and that statistical flow properties such as velocity profiles and migration phenomena can be quantified even in dense systems. This approach opens new experimental possibilities for studying the microscale dynamics of opaque multiphase flows across a wide range of scientific and technological domains.

## Experimental method

### Overview of experiment

**Fig. 1:**
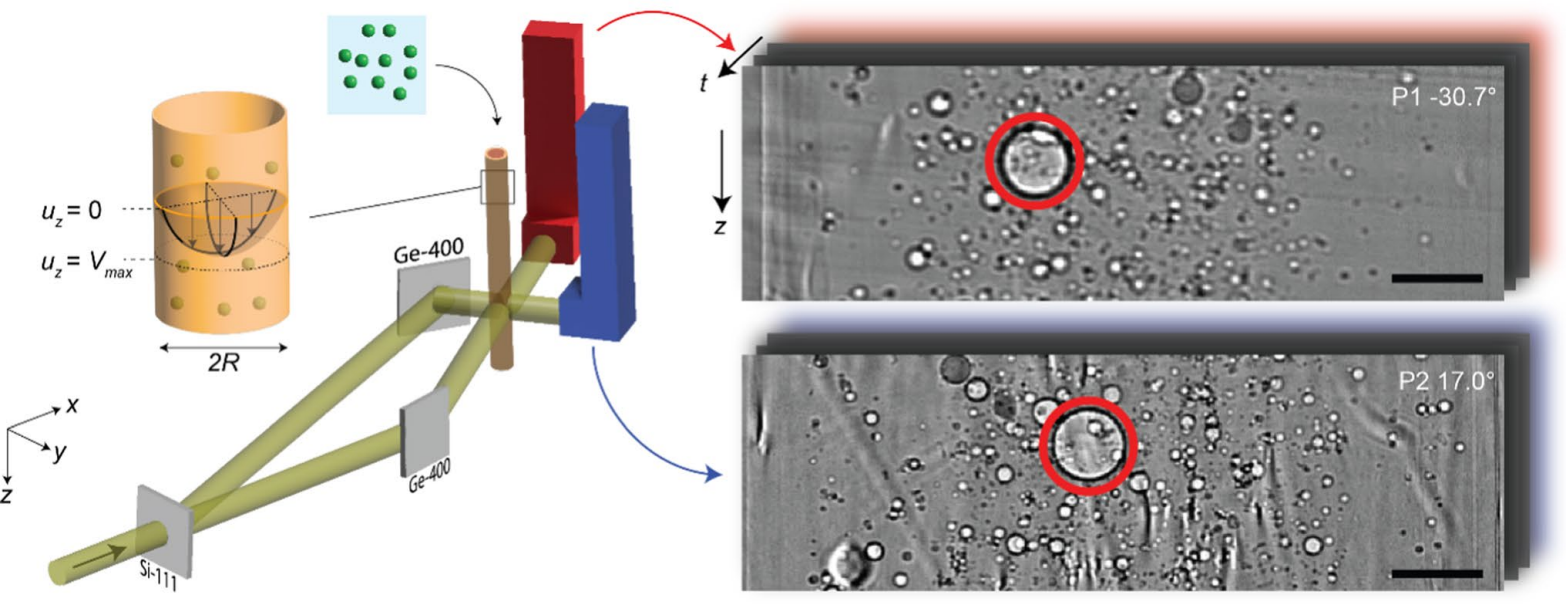
Experimental setup and XMPI concept for multiphase flow experiments. The direct beam is split and recombined to produce two stereographic projections, enabling 4D tracking of individual particles from two directions. The red circle highlights a large particle seen in both directions. In two projections, different performances of edge enhancement of particles are observed due to slightly different sample-detector distances. Scale bars: 100 µm. Angles related to the direct beam direction. See Movie S1 for the recorded movie

The XMPI experiment was performed at the ForMAX beamline, MAX IV, Lund, Sweden. Photos of the experimental setup are provided in Fig. 8 in Appendix A. The direct beam was split using the Bragg reflection of Silicon and Germanium crystals (Fig. 1). The X-ray photon energy was 16.55 keV, with two beamlets at an angle of approximately 48°, simultaneously illuminating the sample. The transmitted intensity from each beam was recorded by two X-ray microscopes with an effective pixel size of 1.3 µm, positioned at an intermediate distance (several centimeters) after the sample. In principle, larger sample-detector distances are beneficial for observing low absorption-contrast samples at the cost of reducing the accuracy of determining the locations of high absorption-contrast particles; while smaller sample-detector distances are beneficial for samples with high absorption-contrast. The propagation distance we used can help to observe some low absorption-contrast features due to phase contrast (Snigirev et al. 1995; Suzuki et al. 2002) without compromising the capability of locating particles. Note that the slight difference in propagation distances between the sample and the two microscopes leads to a visible difference in propagation effects in the two projections, which could not be further improved due to the setup constraints during the presented experiments. The sample consists of a Kapton tube with an inner diameter of 2*R* = 0.72 mm with a flow driven by a syringe pump. To highlight this technique’s potential, we demonstrate two multiphase flow scenarios, with movies provided as Supplementary Material:

- Suspended microparticles in glycerol.
- Suspended microparticles in human blood.

### Sample

Choosing suitable particles with sufficient contrast for X-ray imaging experiments of flowing suspensions needs careful considerations. X-ray contrast is higher for heavier elements, but high-density particles can cause significant sedimentation and inertial effects, which is problematic if the goal is to trace the motion of the fluid. In phase-contrast X-ray imaging, hollow particles are easily detectable as the gas–solid interface causes much lower intensity values than absorption-contrast image for solid tracers (Kim et al. 2009). Furthermore, the hollow nature can aid in reducing the particle density to remain close to neutrally buoyant, and edge contrast can be further enhanced by metal coatings (Parker et al. 2022). The particles used in this work were silver-coated hollow borosilicate glass spheres (SHGS, Dantec Dynamics) with density *ρ* = 1400 kg/m^3^. As a suspension media, we used glycerol with a density of* ρ*_s_ = 1260 kg/m^3^ and viscosity *µ* = 1.4 Pa·s. The diameters range between 5 and 35 µm with mean diameter 2*R*_p_ = 10 µm. Figure 1 illustrates a sequence of frames at a concentration of 1 wt % (0.9 vol.%). These particles, commonly used as tracers in fluid dynamics research, are well-suited for X-ray imaging (Parker and Mäkiharju 2022). Due to their hollow nature, they appear with lower attenuation than the surrounding fluid and an interface enhanced by phase-contrast imaging.

Human whole blood of about 40% hematocrit up for destruction, e.g., expired, was used for the experiments. The blood was obtained from the Department of Transfusion Medicine at Karolinska University Hospital, Huddinge, Sweden.

### Flow

The flow was driven with a syringe pump (New Era Pump Systems NE-4000) with a 1 mL syringe at a set flow rate of *Q* = 0.1 mL/h through a Kapton tube (Allectra 312-KAP-TUBE-07-300), with a reported inner diameter of 2*R* = 0.72 mm and 25 µm wall thickness, leading to a theoretical maximum velocity of *V*_max_ = 2*Q*/(*πR*^2^) = 0.137 mm/s. The distance from the flow entering the Kapton tube to the measurement section is approximately 10 mm.

The theoretical sedimentation velocity is *V*_sed_ = 2(*ρ*−*ρ*_*s*_)*gR*_*p*_^2^/(9*µ*) ≈ 5.45 × 10^−6^ mm/s, and since *V*_sed_ ≪ *V*_max_, we can assume particles at a radial position r from the center of the capillary are following the analytical Poiseuille velocity profile *u*_*z*_ = (2*Q*/(*πR*^2^))(1−*r*^2^/*R*^2^). This equation is used to fit the experimental data, using *Q* and *R* as fitting parameters, with residuals defined by the error in radial position *r* for a certain measured velocity *u*_*z*_.

From the experimental data in this work, we found that the actual inner diameter was 2*R* = 0.78 mm and the actual flow rate *Q* = 0.115 mL/h, leading to a maximum velocity of *V*_max_ = 0.134 mm/s. The Reynolds number of this flow is Re = *ρ*_*s*_* · *(*V*_max_/2) · 2*R/µ* = 4.7 × 10^−5^.

### X-ray multi-projection setup

The experiments were performed at the ForMAX beamline at MAX IV (Nygård et al. 2024; Tavares et al. 2014), the first operational diffraction-limited storage ring. The ForMAX beamline is in the MAX IV 3 GeV storage ring, utilizing undulators as source. At this beamline, we performed the experiments at a photon energy of 16.55 keV, which delivers one of the highest photon fluxes over a narrow bandwidth (Δ*E*/*E *≈ 0.01, provided by the multilayer monochromator) and an illumination size of 1 × 1 mm^2^ with a collimated beam (Yao et al. 2024). Using the direct beam, we used the crystal configuration shown in Fig. 1, inspired by Mokso and Oberta (2015) to generate the two beamlets to perform Particle Tracking Velocimetry (PTV). The first beamlet was split from the incoming beam by using a Si-111 crystal that generated a deflection angle with respect to the main beam of −13.72°. This beam was recombined into the sample position by using a Ge-400 crystal, which generated a recombination angle of 16.99° with respect to the main beam (P2 in Fig. 1). The second beamlet was generated by using a Ge-400 crystal, which generated a beam deflection of −30.70° (P1 in Fig. 1). Thus, the angle between the two beamlets used for PTV was 47.69°. Although a 90-degree angle separation is optimal for PTV studies as shown in Appendix B and Fig. 11b, it is important to note that the selection of 47.69° is based on a compromise between the diffraction efficiency (1–2%) and the angular separation, ensuring both proper angular range and image quality provided by the XMPI setup (Bellucci et al. 2024; Rogalinski et al. 2026). The sample was positioned at the intersection point between the two beamlets. The two beamlets were detected with two identical indirect X-ray microscopes. The X-ray microscopes used a LuAG:Ce scintillator and a high-NA 5X magnification lens to provide an effective pixel size of 1.3 µm when coupled to an Andor Zyla 5.5 sCMOS camera. The setup was operated at 40 Hz, and the exposure time was set to the inverse frame rate to maximize the contrast-to-noise ratio. Given this configuration and experimental setup, we find that blurring of particle edges starts occurring when the particles move more than ~ 2 pixels during exposure, i.e., around 0.1 mm/s. To avoid blurring at this frame rate, the exposure time can be reduced at the cost of decreasing the contrast-to-noise ratio, with the field-of-view limiting the maximum velocity. Assuming a maximum allowed displacement of half the field-of-view (~ 500 µm) to identify particles in two consecutive frames, the maximum velocity that can be tracked is 20 mm/s in the current setup. Faster flows can be studied with kHz and beyond cameras, for example, using Photron Nova S16 (16 kHz), as previously used (Asimakopoulou et al. 2024) and currently available and commissioned at the ForMAX beamline in MAX IV (Rogalinski et al 2026, and Witte et al. 2026). Such a setup has the potential to probe particle velocities of 8000 mm/s, and thus providing a theoretical possibility to study Reynolds numbers of 4000 with this change of cameras for a suspension in water.

### Image preprocessing

Analogous to conventional flat-field correction (Van Nieuwenhove et al. 2015), the recorded images (*I*_raw_) by both cameras were corrected to eliminate fixed pattern noise due to the illumination and the detector's dark current. For that, we acquired 100 glycerol-only images (by applying a flow of glycerol with a high flow rate to avoid residual particles in the field-of-view) and 100 dark-current images (images without illumination) for every experiment. The corresponding averaged glycerol-only images (*I*_B_) and averaged dark-field images (*I*_D_) were used for correction, using the formula *I*_corrected_ = (*I*_raw_−*I*_D_)/(*I*_B_−*I*_D_).

## Analysis methods

### Analysis of dilute suspensions

#### 4D Microparticle tracking

**Fig. 2:**
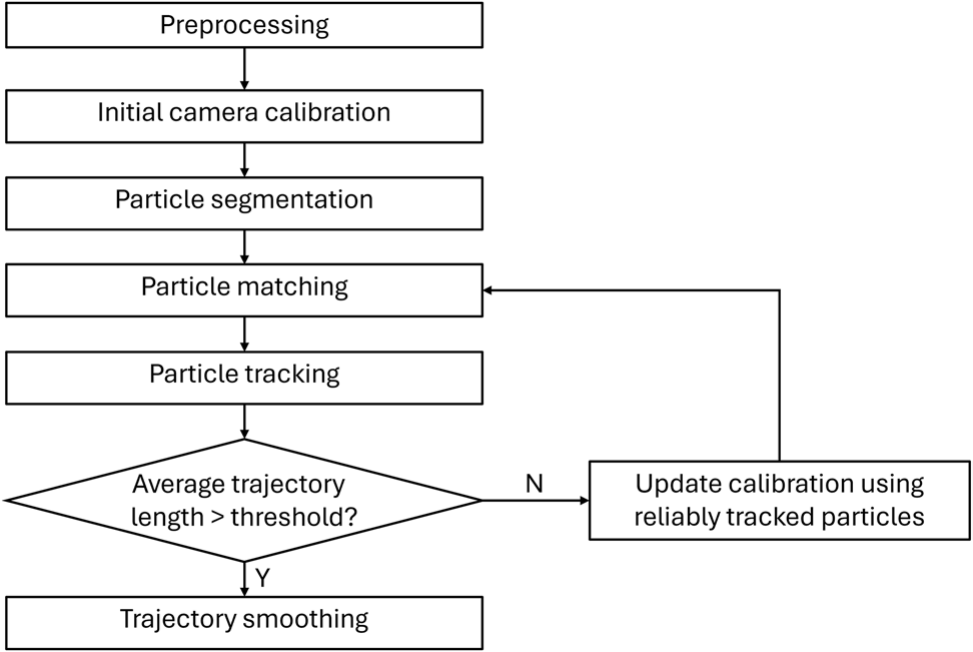
Flow chart of MyPTV-based microparticle tracking

Each XMPI experiment consisted of 8000 frames simultaneously recorded on each of the two detectors at 40 Hz. In 4D microparticle tracking, we aim to get the 4D trajectory of each particle as well as their velocity. The flowchart is shown in Fig. 2, including image preprocessing and an iterative particle tracking workflow based on MyPTV (Shnapp 2022), an open-source Python-based software. Specifically, there are two main purposes for image preprocessing. The first is to create a calibration target for PTV using the projected positions of one manually tracked particle; the second is to help with the particle segmentation and detection. In the initial calibration step, we created a pinhole camera model (see Appendix B for details) with the help of the calibration target created from the preprocessing step and the angular configuration of the XMPI setup. In the particle segmentation step, we detected the centroids of each particle for every frame in both cameras. In the particle matching step, particles detected in both cameras are matched so that their 3D positions can be triangulated with epipolar geometry. In the particle tracking step, we linked the matched particles in the time domain to form their 4D trajectories. Reliably tracked particle trajectories are then used for updating the camera model. The steps of particle matching, particle tracking and camera model updating follow an iterative process, until the average temporal trajectory length reaches a certain level after the particle tracking step. After getting the 4D trajectories, linear fitting is implemented to calculate the particle velocity, as done in the trajectory smoothing step. More technical details on each step are available in Appendix B.

#### Error analysis

In the workflow presented in Sect. 3.1.1, there are several sources of uncertainty to consider when determining particle positions. First, in the current XMPI setup, the instability of each beamlet results in a slight movement of the field-of-view, as can be seen from Movie S1 to S5. Such a movement is usually within 1 pixel. Second, in the particle segmentation step, as stated in Nyquist sampling theorem, the minimum resolvable feature size from an image is 2 pixels. Therefore, when we regard the centroid of a particle as a feature, an uncertainty of 1–2 pixels should be taken into account. Considering the difficulty of deciding the center of each particle, especially when several particles contact or interact with each other, a reasonable estimation of such uncertainty is 2 pixels. Third, the camera calibration process can result in a 1-pixel uncertainty, as suggested by MyPTV documentation (Shnapp 2022). When determining particle velocities, we need to consider both the uncertainty of particle position and the linear fitting process. As a result, in this study, the uncertainty of detecting vertical (radial) position is determined as 2.45 (4.7) pixels. The uncertainty of vertical velocity (at the scale of ~ μm/s) depends on the temporal length of the trajectory. A longer trajectory results in a lower uncertainty. The derivations of the error analysis are provided in Appendix D.

Although the presented error analysis is performed for this specific task of tracking particles in dilute suspensions using XMPI, it can also be extended or modified for other scenarios. For example, when studying multiphase flow systems with a larger particle concentration, or when streaking effects are observed on fast-moving particles, segmenting and determining centroids of the particle can be even more challenging, which results in an uncertainty larger than 2 pixels. When tracking particles in more complex flows (e.g., turbulent flows), linear fitting will be replaced by polynomial fitting for calculating the velocity, which means that the error analysis (Eq. 8) needs to be modified accordingly.

### Analysis of dense suspensions

At dense concentrations of SHGS particles with constant particle overlaps, it is not possible to match individual particles in the stereographic views. However, the penetration capability of X-rays allows for integrated fluid motion to be captured over the path of the beam. Even though there is limited information to fully resolve the 3D flow field in time, an additional simultaneous projection can be used to spatially determine certain flow features, e.g., regions of recirculation, and provide valuable data for comparisons with numerical simulations.

In this particular work, since an axisymmetric flow was used with no expected differences between the two projections, we only use this to exemplify how an XMPI experiment of dense suspension flows can be analyzed with any common technique for image velocimetry. Specifically, we show that we can recover the expected projected velocity profile from both projections, using simple common flow analysis techniques such as Optical flow (OF) and Particle Image Velocimetry (PIV).

Optical flow (OF) analysis was done using opticalFlowRAFT in MATLAB R2024b (MathWorks 2024). This function estimates the velocities of each pixel between consecutive video frames using the recurrent all-pairs field transforms (RAFT) algorithm (Teed and Deng 2020; Lagemann et al. 2021) via a deep-learning network trained on the Kubric dataset (Greff et al. 2022). The velocities were scaled using the known pixel size and frame rate, then averaged over time and vertical direction. Only default settings of the function were used for flow estimation. In OF, projected velocity vectors are assigned to every pixel in the image.

Particle Image Velocimetry (PIV) was performed on the same data using PIVlab v3.02 (Thielicke and Sonntag 2021) in MATLAB R2023b. The PIV algorithm used FFT window deformation with one pass using an interrogation area of 32 pixels and a step size of 16 pixels. In PIV, projected velocity vectors are assigned to every interrogation area of the image.

The main difference between the two methods is that PIV tracks particle pattern displacement in an interrogation area via correlation, while OF infers motion from image intensity changes of single pixels via mathematical constraints and learned priors from known training data (Lagemann et al. 2021). In a system of large overlapping particles, we expect OF to be more suitable as we will track the motion of an intensity pattern rather than individual tracer particles.

Spatial horizontal coordinates in the two projections are determined from projected wall positions, which were clearly visible in the raw images. In order to avoid erroneous vectors from image noise, we define a measurement window 50 pixels away from the walls where there are particles present throughout the image height.

## Results

### 4D tracking of individual microparticles flowing in glycerol

**Fig. 3:**
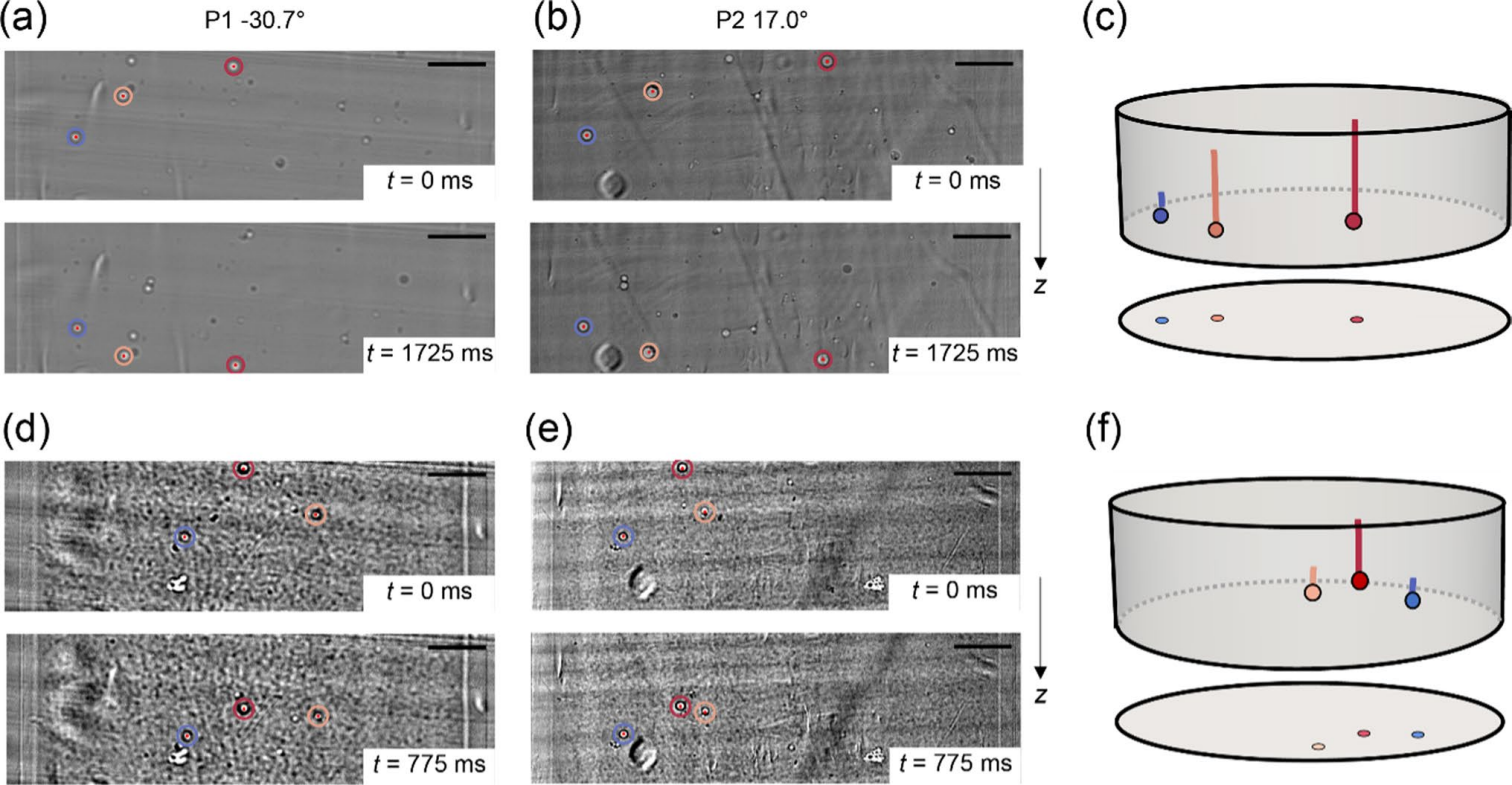
4D particle tracking of microparticles. Three SHGS particles (colored rings) flowing in glycerol (**a**, **b**) or blood (**d**, **e**) were identified in two projections at two-time instances with red dot indicating the tracked particle center position. Reconstructed 4D trajectories between these two-time points for glycerol (**c**) and blood (**f**). Scale bars: 100 µm. Datasets for **a–b** and **d–e** are in Movie S2 and Movie S3, respectively

Firstly, we studied a laminar flow of polydisperse silver-coated hollow-glass spheres (SHGS) in glycerol. At low concentrations, each particle was distinguishable in both projections by vertical coordinate *z* and velocity. Tracking can be further verified by the polydispersity of particles, as they are distinguishable by size. Figure 3 shows the 3D positions of each particle for a given time range. For example, three different particles are matched and tracked between two different times in Fig. 3a, b, which was performed using a calibrated coordinate system and the MyPTV library (Shnapp 2022). By tracking the particles over time in 3D, we resolved their 4D trajectories, as depicted in Fig. 3c. The Appendix C includes a detailed trajectory analysis in Fig. 10a, b.

### 4D tracking of individual microparticles flowing in human blood

As a second case, we studied the motion of SHGS particles in human blood as an example of a highly dense suspension. Although blood is opaque to visible light, the microparticles were easily distinguishable in our experiment. Using the aforementioned procedure, we identified particles in two projections, Fig. 3d and e, allowing us to retrieve their 3D position and track them over time. The 4D trajectories of three particles in blood are depicted in Fig. 3f, with a detailed trajectory analysis in Fig. 10c, d of Appendix C. Although red blood cells (RBCs) were not directly visible, a speckle pattern in both projections, see Fig. 3d and e, was observed. Such a pattern has been previously used to track blood flow with X-rays in individual 2D projections (Kim and Lee 2006; Lee et al. 2010; Irvine et al. 2008).

### Retrieving statistical properties in dilute and concentrated suspensions

These experiments demonstrate the ability to track individual micrometer-sized particles in 4D within opaque multiphase flows using XMPI, enabling the study of stochastic phenomena not captured by techniques like OCT or MRV. Although this method can be used to track individual particles, it can also be used to study statistical properties. For instance, as small particles primarily follow the fluid motion, we can perform statistical 3D particle tracking velocimetry (3D PTV) of these trajectories, providing velocity fields comparable to other methodologies.

#### Particle tracking velocimetry (PTV) in dilute suspensions

**Fig. 4:**
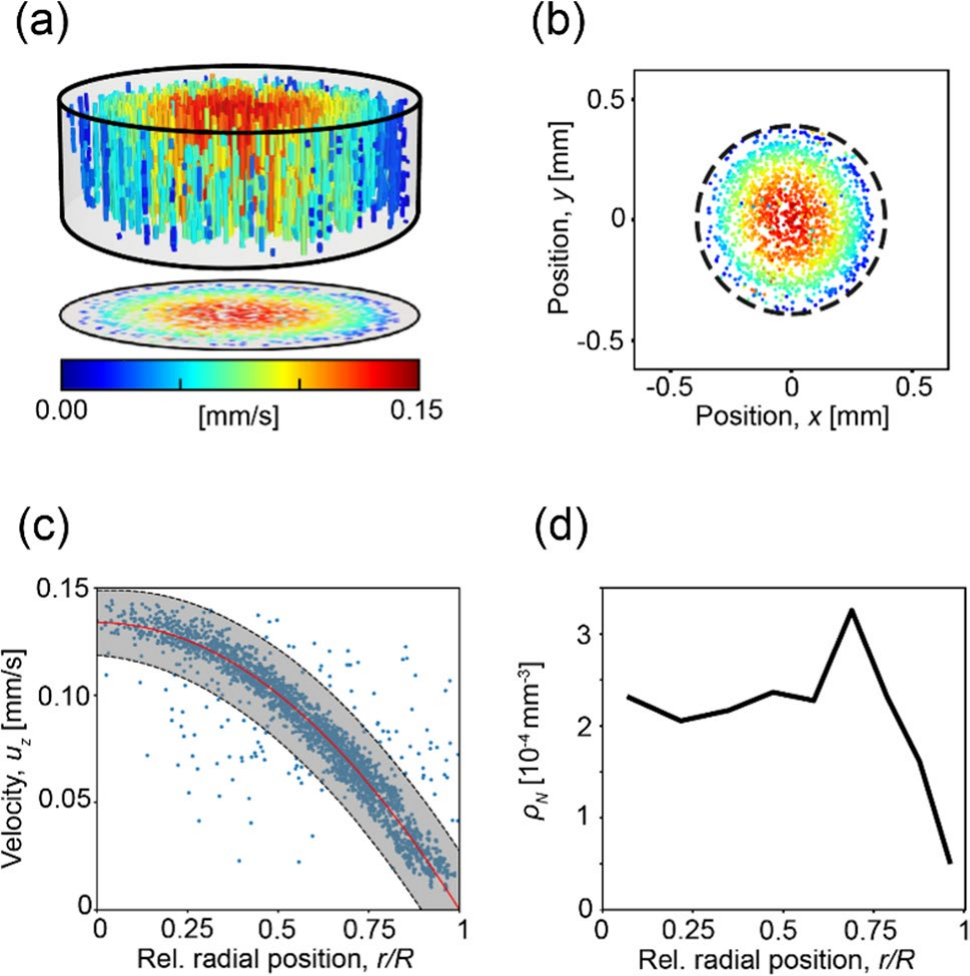
Statistical analysis of dilute SHGS particles in glycerol using 3D PTV. **a** Trajectories of 2471 tracked particles at 0.1 wt % concentration, hue indicates vertical velocity *u*_*z*_. **b** Horizontal distribution of particles and their velocities *u*_*z*_ using the same hue scale. **c** *u*_*z*_ as a function of radial position *r* compared with theoretical cylindrical Poiseuille flow profile (black curve). **d** Particle number concentration *ρ*_*N*_ as a function of radial position. Datasets for PTV are provided in Movie S4. A rendering of the 4D data set in **a–c** is provided in Movie S6

To demonstrate the ability to retrieve statistical properties and validate the XMPI approach, we tracked 2471 SHGS particles (with a mean diameter of 15 μm and a standard deviation of 3 μm) in glycerol flowing through the cylindrical capillary, as shown in Fig. 4a. We further determined their average horizontal positions and downstream velocity as a function of radial position, as shown in Fig. 4b and c, respectively, where the horizontal location with the highest downstream velocity was marked as the origin of the horizontal plane. In Fig. 4c, a 3σ error band was estimated by considering both uncertainties of radial position (6.1 μm) and velocity (0.005 mm/s), where details can be found in the Appendix D and Fig. 11. As a result, 95.8% of all tracked particles were inside the error band. Both radial velocity and concentration profiles were extracted from low-density suspensions (0.1 wt %), as higher concentrations complicate such studies due to challenges in detecting particle centers when multiple particles are in contact within the camera view. The figure also shows that the resulting particle velocities fit accurately to the expected laminar Poiseuille flow profile. Plotting the number density of particles *ρ*_*N*_ versus radial position as seen in Fig. 4d, there are clearly fewer particles in the direct vicinity of the walls. Although this is expected owing to the Segré–Silberberg effect (Segre and Silberberg 1961, 1962; Di Carlo et al. 2007), it is unlikely that such “inertial focusing” is visible at these creeping flow conditions, and the particle-free layer likely stems from non-ideal upstream conditions and potential fouling of the tube by near-wall particles. It is still worth emphasizing the possibility of studying general migration phenomena, including inertial focusing, using XMPI and 3D PTV in opaque systems.

#### Effect of flow rate

**Fig. 5:**
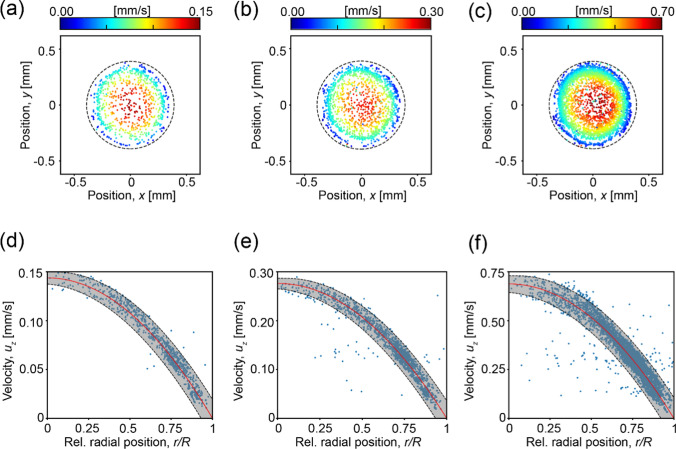
Microparticle tracking at a particle density of 0.05 wt at three different flow rate settings **a** 0.1 mL/h, **b** 0.2 mL/h, and **c** 0.5 mL/h. The lower figures **d–f** show the particle velocities as function of radial positions at the same concentrations, where the shadowed region indicates the 3σ error band. The proportions of tracked particles within the error band are 97.3%, 95.7%, and 96.3%, respectively

Figure 5 shows the results at the same particle density of 0.05 wt % at three different flow rate settings (0.1 mL/h, 0.2 mL/h, 0.5 mL/h), respectively. The first row a–c shows the particle velocity distribution in different horizontal positions and the second row d–f shows how the particles follow the expected theoretical velocity profile of an incompressible laminar flow through a cylinder (solid curve). The proportions of tracked particles within the error band are 97.3%, 95.7%, and 96.3%, respectively. Since the total acquisition time is the same for all the flow rates, we naturally are able to track a larger number of particles at the higher flow rate. The number of tracked particles are 933, 1549 and 4596, respectively, for the three flow rates. The number of tracked particles is roughly five times higher at five times higher flow rate, indicating that streaking of particles during exposure is less of an issue within the experimental range here. According to the theoretical velocity profile, the maximum velocity at 0.5 mL/h should be close to 0.7 mm/s, i.e*.* approximately 13.5 pixels during an exposure of 1/40 s. Most of the fastest particles would thus have moved on the order of a particle diameter during the exposure, and likely are blurred. Nevertheless, many particles can be tracked at these velocities despite such streaking effects as seen in Fig. 5c and f. It still remains a sensible strategy though to limit motion to 2–3 pixels per exposure and set the flow rate according to the necessary exposure time and frame rate.

#### Effect of particle concentration

**Fig. 6:**
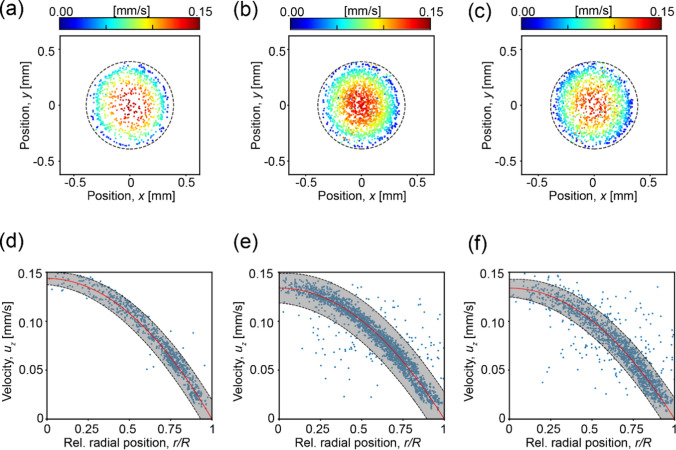
Microparticle tracking at a flow rate setting of 0.1 mL/h; the particle densities are **a** 0.05 wt %, **b** 0.1 wt %, and **c** 0.2 wt %. The lower figures **d–f** show the particle velocities as function of radial positions at the same concentrations, where the shadowed region indicates the 3σ error band. The proportions of tracked particles within the error band are 97.3%, 95.8%, and 90.0%, respectively

Figure 6 shows the results at the same flow rate setting of 0.1 mL/h in three different particle densities (0.05 wt %, 0.1 wt %, 0.2 wt %), respectively. The first row a–c shows the particle velocity distribution in different horizontal positions, and the second row d–f shows how the particles follow the expected theoretical velocity profile of an incompressible laminar flow through a cylinder (solid curve). A 3σ error band is created based on the uncertainties ${\sigma}_{r}$ and ${\sigma}_{{u}_{z}}$ calculated in Appendix D. The proportions of tracked particles within the error band are 97.3%, 95.8%, and 90.0%, respectively. Again, the total acquisition time is the same for all concentrations and the expected number of tracked particles should scale with the concentration. The number of tracked particles are 933, 2471 and 1672, respectively, for the three concentrations. Going from 0.05 to 0.1 wt % follows roughly the expected increase of tracked particles, indicating that the increasing probability of particle overlaps did not significantly influence the results at 0.1 wt %. However, going from 0.1 to 0.2 wt % there is a significant reduction of tracked particles despite there being more particles in the field-of-view, which likely originates from more particle overlaps in the projections. At 0.1 wt % (0.9 vol %), the expected number of 10 μm particles in a center projected particle-wide strip of dimensions 300 × 10 × 780 μm^3^ is approximately *N* = 4. This corresponds to roughly *N*(*N*–1)/2 = 6 possible particle pairs. Since only a fraction of these pairs have the correct ordering and sufficient Poiseuille velocity contrast to overtake within the ROI, this gives an expected crossing count of order unity. Because the number of particles in the strip scales linearly with concentration while crossings are pair events, the crossing count scales approximately as *c*^2^. Here, given our geometry, it seems indeed that a concentration of 0.1 wt % with an expected crossing count around 1 at the center is still acceptable for accurate tracking. However, already at 0.2 wt %, with 4 times higher expected crossing count, there is a significantly lower fraction of particles being tracked using the current framework.

#### Analysis in dense suspensions

**Fig. 7:**
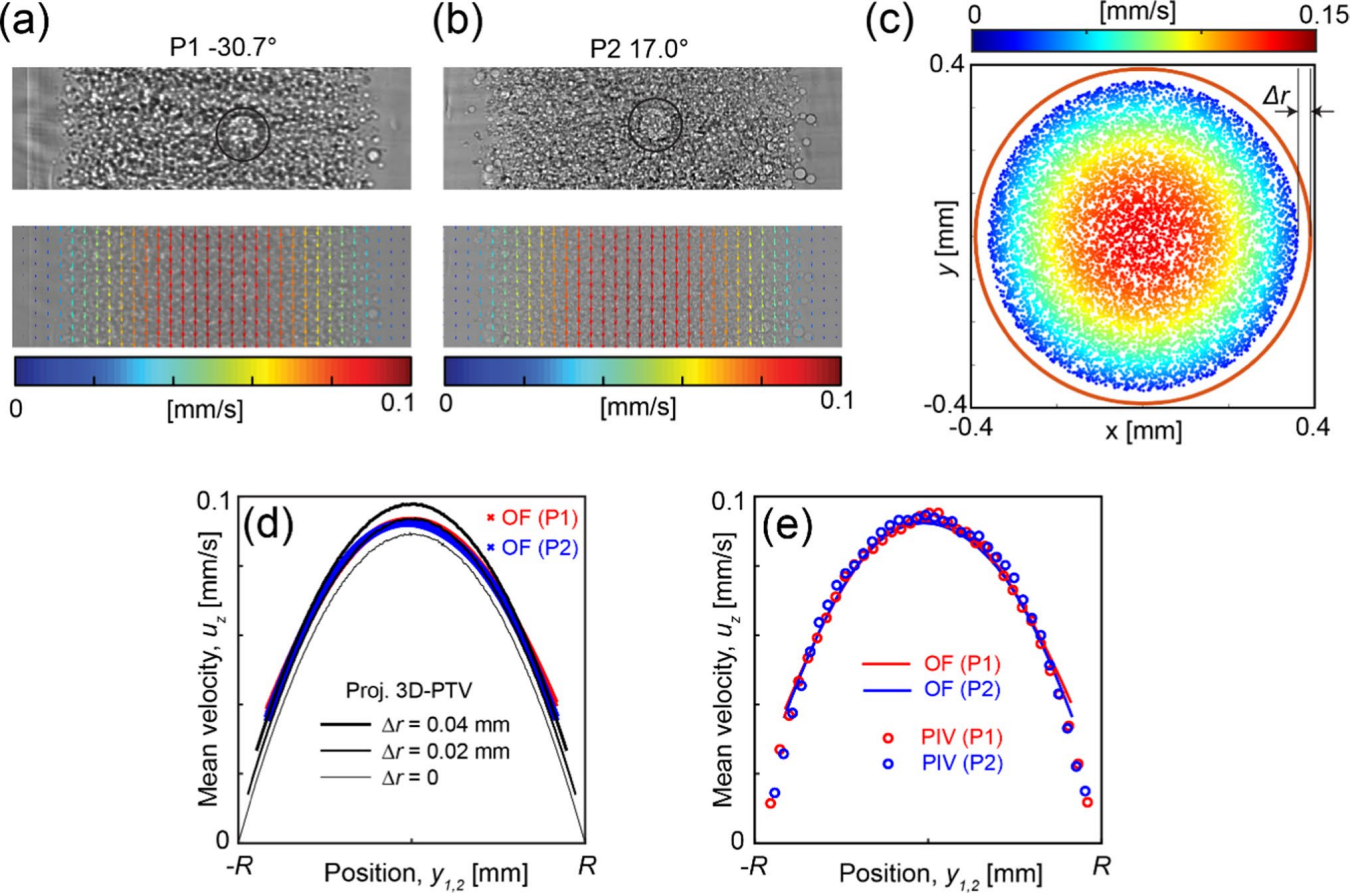
Example analysis of a dense suspension of SHGS particles in glycerol at 10 wt %. (**a**), **b** Projected images P1 and P2 analyzed with optical flow (OF) for each projection. For clarity, only every 20th vector in horizontal and vertical directions are shown. Here, one larger particle can be distinguished in both projections based on size (black circles). **c** Artificial particles are randomly sampled within the tube diameter with velocities assigned from their radial position based on the velocity profile from the 3D PTV experiment, and assuming a particle-free layer of thickness Δ*r* closest to the wall. **d** Comparison of OF results (blue and red crosses) with the projected 3D PTV velocity profile from **c** assuming various Δ*r* (black curves). **e** Comparison of OF analysis and PIV analysis of the same datasets. Datasets for this analysis are provided in Movie S5

**Fig. 8:**
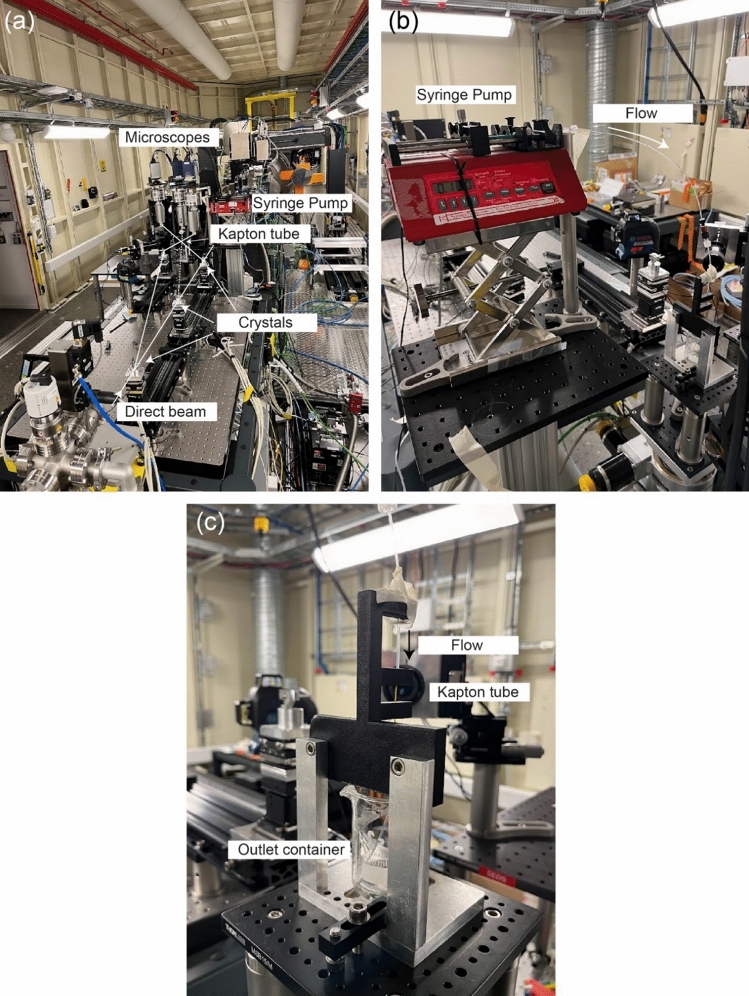
Photos of the experimental setup for XMPI of multiphase flows at the ForMAX beamline, MAX IV, Sweden. **a** XMPI setup; Note that a third crystal and microscope were mounted but not used in this study. **b** Flow setup. **c** Close-up of the measurement section

**Fig. 10:**
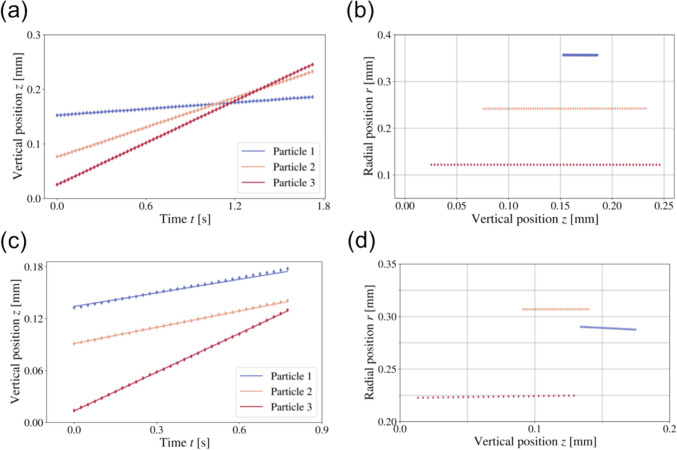
Example of the analysis of three tracked SHGS particles in glycerol (**a**)(**b**) and in blood (**c**)(**d**), respectively. **a** and **c** show how the vertical position changes with time; **b** and **d** show the relationship between the radial position and the vertical position

**Fig. 11:**
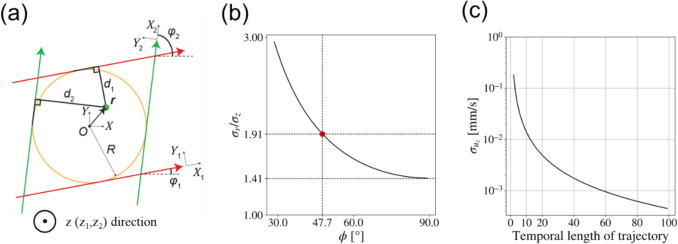
**a** Coordinate system used for error analysis; **b** Relationship between the XMPI angular separation ϕ and the radial position uncertainty described in ratio of ${\sigma}_{r}/{\sigma}_{z}$; **c** Relationship between the temporal length of a specific trajectory and its uncertainty of vertical velocity

Matching individual particles in dense systems from two projections can be challenging, unless the particle can be distinguished through unique spatiotemporal features (e.g., the big particle visible in Fig. 7a and b).

Nevertheless, velocity profiles can be extracted with image velocimetry techniques such as OF and PIV. Both OF and PIV analysis was performed on a 5-s sequence of corrected images (200 images) of SHGS at 10 wt % at 0.1 mL/h, also provided as a supplementary Movie S5. The resulting vector fields from the OF analysis of the two projections are shown in Fig. 7a and b.

At this concentration, suspension viscosity can be assumed to be shear-rate independent and Newtonian. Any increase of effective viscosity only leads to a higher required pressure to maintain the set flow rate on the syringe pump. Thus, the 2D velocity profiles from the OF analysis is expected to be similar to the averaged 3D PTV velocity profile of the dilute case in Fig. 4c projected along the viewing direction.

To test this hypothesis, the expected projected velocity profile is evaluated by randomly sampling 10^7^ artificial particles within a circle with a diameter of 2*R* and assigning them with a velocity according to the fitted data of *Q* and *R* from the 3D PTV results. Since we expect there to be a non-uniform radial distribution of particles (cf. Figure 4d), we investigate the effect of a particle-free layer of thickness Δ*r*. Particles were binned according to their *x*-position, and an average was taken for every bin to provide a comparison to the OF results. The procedure is illustrated in Fig. 7c.

Figure 7d shows the projected velocity profiles from the OF analysis of the dense suspension along with the expected projected velocity profiles from the dilute 3D PTV data at various values of Δ*r*. The fact that the projected profiles from the two measurements are closely matching confirms the cylindrical symmetry of the flow. Without any particle-free layer (Δ*r* = 0), the projected 3D PTV profile is underpredicting the experimental results, but at Δ*r* = 0.02 mm, there is a good agreement, which also is in accordance with expectations. The projected velocity profiles could also be equally well extracted with PIV as demonstrated in Fig. 7e.

The demonstration here shows an indication that the projected velocity profiles of the dense suspensions are captured by image velocimetry of the XMPI images. However, the full validation is out of the scope of this work and further exploration and validation across different dense systems and probes will be considered in future work.

The slight mismatch seen here between the PIV/OF and projected 3D PTV profiles likely arises from the radial concentration of particles not being well represented with a step function. In the dilute case, the radial concentration profile is provided in Fig. 4d, but the concentration profile is likely different for the dense suspension flow. To further improve the results, a measurement without phase-contrast edge enhancement could lead to an attenuation-based projected concentration profile. Complementing the mean projected velocity determination with such measurements, assuming cylindrical symmetry and no swirl, the radial concentration profile and 3D velocity profile can be recovered via an Abel transform, which can be done in a single projection.

Ultimately, having two views further allows us to assess e.g*.* cylindrical symmetry and quantify projected flow fields without this symmetry. Even though there is not enough information to directly find the flow field through, e.g., Abel inversion, the demonstration here shows how projected profiles can be matched between simulations and experiments. By testing various scenarios and parameters with a CFD model and matching projected profiles, the simulation can be used as a digital twin to illustrate most likely 3D velocity fields that would generate the projected velocity and concentration profiles in the experiment. Full 3D flow field determination can also be enabled through more projections using PIV techniques as demonstrated by Dubsky et al. (2010) using 9 angularly spaced projections.

## Discussion and conclusions

We demonstrated XMPI’s ability to be used for multiphase flow research by tracking micron-sized particles or other microscopic features in 4D in flows without sample rotation. The method can furthermore be expanded to studies of flows through complex geometries, e.g., porous media or even custom-made micro-3D-printed geometries. This method provides otherwise inaccessible experimental validation for particle-resolved computational fluid dynamics (CFD) simulations. With more projections, XMPI might be used to capture the instantaneous full 3D velocity field of dense suspensions with 3D PIV, as described by Dubsky et al. (2010). This could be done directly on the speckle pattern from blood (Kim and Lee 2006; Irvine et al. 2008; Lee et al. 2010), allowing a detailed comparison of particle trajectories and simultaneous velocity fields of RBCs in 4D. In dense suspensions of solid particles, XMPI can reveal microstructural dynamics leading to aggregation, yielding, and jamming (Ness et al. 2022; Stickel and Powell 2005), crucial for understanding the suspension rheology. As we emphasize the experimental modality here using easily accessible open-source packages for particle tracking and OF/PIV, there can also be a further improvement to the analysis of the acquired images allowing for 4D particle tracking at concentrations above dilute conditions.

Beyond particle suspensions and blood flow, XMPI is also well suited for a broader range of multiphase systems involving fluid–fluid interfaces, such as bubbly flows, liquid foams, and emulsions. In these systems, the same propagation-based phase-contrast mechanisms that make engineered hollow tracers highly visible also strongly enhance naturally occurring gas–liquid interfaces, since the abrupt change in electron density at bubble surfaces generates pronounced edge contrast. This provides a favorable basis for tracking bubble motion, deformation, coalescence, breakup, and collective dynamics in opaque geometries, while extending the method to liquid–liquid dispersions (i.e., emulsions) where droplets can be resolved when sufficient density or compositional contrast exists between the phases. Practical limitations remain linked to the choice of continuous phase medium: highly absorbing liquids can reduce transmitted intensity and signal-to-noise ratio, while phases with electron density too similar to the dispersed phase may yield insufficient interfacial contrast, requiring optimization of X-ray energy, propagation distance, or contrast agents.

Despite the potential of micrometer-resolved XMPI for multiphase flows at synchrotron light sources, there are limitations worth mentioning. The limited field-of-view (currently < 10 mm), limited angular separation (currently 47.69°), and the availability of these facilities render many experiments more suitable for lab sources, especially when high spatiotemporal resolution (micrometer resolution with ~ 100 Hz or higher frame rates) is unnecessary. Although the temporal resolution in this experiment was modest (40 Hz), higher frame rates are achievable using faster X-ray detection schemes. The ultimate achievable spatiotemporal resolution depends on flux density, i.e., the number of photons per unit of time and area on each beam. Diffraction-limited storage rings (e.g., MAX IV Tavares et al. 2014, ESRF-EBS Raimondi 2016, and APS-U Kerby 2023) and X-ray free-electron lasers (e.g., EuXFEL Decking et al. 2020, LCLS Emma et al. 2010) increase the flux density by more than one order of magnitude compared to the previous generation of synchrotron light sources, enabling micrometer resolution with kHz framerates and beyond (Asimakopoulou et al. 2024; Villanueva-Perez et al. 2023). Higher flux also offers opportunities for more beamlets and, thus, higher angular resolution. Combining XMPI and 4D XCT reconstructions from sparse projections using machine learning (Zhang et al. 2024) suggests a future where high-speed, high-resolution imaging of multiphase flows without sample rotation becomes feasible.

To summarize, we report the first experiment of a multiphase flow using XMPI at a synchrotron light source, achieving time-resolved micrometer-scale 4D imaging of opaque fluids, such as blood. We demonstrated the possibility of tracking individual particles in 4D and the possibility of determining statistical flow properties through 3D PTV such as the velocity profile and migration behavior. We also quantified instantaneous projected velocities in dense suspensions using OF and PIV. Thus, we envision that this methodology will open up a new frontier in experimental studies of multiphase flows, with applications not limited to particle suspensions but also extending to gas–liquid flows and immiscible liquid–liquid flows.

## Electronic supplementary material

Below is the link to the electronic supplementary material.

Supplementary file1 (MP4 4257 KB)

Supplementary file2 (MP4 7360 KB)

Supplementary file3 (MP4 9550 KB)

Supplementary file4 (MP4 5170 KB)

Supplementary file5 (MP4 4679 KB)

Supplementary file6 (MP4 29071 KB)

## Figures and Tables

**Fig. 9:**
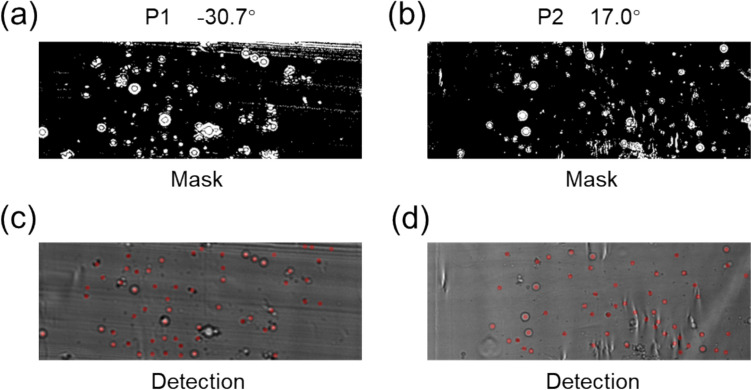
Examples of particle segmentation in both cameras. **a** and **b** show the binary mask of moving objects created in the preprocessing step for both cameras, respectively. **c** and **d** show the detected particle positions (red dots) in both cameras after the particle segmentation step

## Data Availability

Data will be made available on request.

## Appendix A: Photos of experimental setup

Photos of the XMPI experimental setup at the ForMAX beamline at MAX IV, are provided in Fig. 8.

## Appendix B: Details of MyPTV-based microparticle tracking

The microparticle tracking procedure includes two preprocessing steps and several main steps using MyPTV toolbox.

In the first preprocessing step, we generated calibration images and the calibration target file for the initial camera calibration implemented later in MyPTV. First, we manually tracked one particle in both views whose 2D trajectory was parallel to the wall. Such a manual tracking can be validated by the vertical position and the size of the particle, as illustrated in Fig. 3. We selected ten frames with a constant stride when this particle was within the field-of-view of both cameras. Second, we generated the calibration image for both views by summing up these ten frames so that ten different locations of the particle were clearly seen in both views. Third, we created the calibration target file by marking the *z* positions of the particle. The horizontal position ***r*** = (*x,y*) was set as (0, 0) temporarily for this step and will be determined later based on the tracking results.

In the second preprocessing step, we created a binary mask of moving objects to help MyPTV detect the particles more efficiently. The mask was created by thresholding pixels that differ by a certain percentage from the mean image taken of the entire set of flat-field corrected frames (5% and 7.5%, respectively, for the two detectors). Some problematic regions of the images with fluctuating pixel intensities were manually determined and removed from the binary mask. Examples of such binary masks for both cameras are shown in Fig. 9a and b, respectively.

The main steps using MyPTV are described as follows:

1. Initial camera calibration

The pinhole camera model used in MyPTV toolbox is depicted in Eq. (1), where $\overrightarrow{r}$ and $\overrightarrow{O}$ denote the particle position and camera’s center position in lab-space coordinates, respectively; η and ζ denote the particle position in 2D image space coordinates; ${x}_{h}$ and ${y}_{h}$ denote the displacement to camera’s center position; $\left[R\left({\theta}_{1},{\theta}_{2},{\theta}_{3}\right)\right]$ is the rotation matrix, which is a function of three orientational angles in 3D space; $\overrightarrow{e}\left(\eta , \zeta \right)$ denotes a nonlinear correction term controlled by ten coefficients ${E}_{11},{E}_{12}\dots {E}_{25}$, which is assumed to be a quadratic polynomial of η and ζ, as shown in Eq. (2).

1 $$\overrightarrow{r}- \overrightarrow{O}= \left.\left(\left[\begin{array}{c}\eta +{x}_{h}\\ \zeta +{y}_{h}\\ \left|\overrightarrow{O}\right|\end{array}\right]+ \overrightarrow{e}\left(\eta , \zeta \right)\right.\right)\cdot \left[R\left({\theta}_{1},{\theta}_{2},{\theta}_{3}\right)\right]$$

2 $$\overrightarrow{e}\left(\eta , \zeta \right)=\left[\begin{array}{c}\begin{array}{c}{E}_{11} \\ {E}_{21} \end{array}\\ 0\end{array}\begin{array}{c}\begin{array}{c}{E}_{12}\\ {E}_{22}\end{array}\\ 0\end{array}\begin{array}{c}\begin{array}{c}{ E}_{13}\\ { E}_{23}\end{array}\\ 0\end{array}\begin{array}{c}\begin{array}{c}{ E}_{14}\\ { E}_{24}\end{array}\\ 0\end{array}\begin{array}{c}\begin{array}{c}{ E}_{15}\\ { E}_{25}\end{array}\\ 0\end{array}\right]\cdot \left[\begin{array}{c}\eta \\ \zeta \\ {\eta }^{2}\\ {\zeta }^{2}\\ \eta \zeta \end{array}\right]$$

When conducting the experiments, a suitable calibration target for our X-ray imaging setup (analogous to, e.g., a chessboard for visible-light systems) was not available. Therefore, in the initial camera calibration, the calibration images generated in the first preprocessing step are used as a “substitute calibration target”. Although ten particle positions are generally not enough for a precise calibration, we can still get a reasonable initial camera model because the angles of two detectors in the XMPI setup are precisely known due to the diffraction condition of the used splitters. This step is done once we get the two camera models that satisfy the angular configuration of the XMPI setup with an averaged calibration error of less than 1 pixel for all ten particles shown in the calibration images. It is worth mentioning that such initial camera models do not contain the nonlinear correction term $\overrightarrow{e}\left(\eta , \zeta \right)$, which will be modified during step 5.

2. Particle segmentation

The masked images from the second preprocessing step are used as the input of this step. We use a local mean subtraction filter, a median noise removal filter, and a Gaussian blur filter provided in MyPTV to boost the performance of particle segmentation. The output of this step is the centroids of each particle with a diameter between 3 and 20 pixels in both cameras. Examples of particle centroids detection for both cameras are shown in Fig. 9c and d, respectively.

3. Particle matching

At this step, segmentation results are used to triangulate the 3D positions of the particles. A prerequisite for such triangulation is to match particles in both cameras. However, brutal search from the camera space is impractical due to a large number of possible “particle pairs”. Therefore, MyPTV provides “particle marching” algorithm to address this issue in a reverse manner. It begins by assuming we have initial guesses of particle positions in lab-space, and then check whether these initial guesses correspond to physical particles in images from both cameras using the co-linearity condition. If so, the correct locations are stored and used as the initial guesses for the next time point. By looping forward from the first time point to the last time point and looping backward from the last time point to the first point, we achieve the 3D position of each successfully matched particle. Practically, the most important parameter in MyPTV at this step is the maximum allowed triangulation error in lab-space coordinates. In this study, we set this parameter to the length of 3–5 pixels.

4. Particle tracking

The 3D trajectories of the particles are formed by linking particles from step 3 in the time domain. At this step, we use the nearest neighbor method (Ouellette et al. 2006) provided in MyPTV.

5. Calibration with reliably tracked particles

With the camera models obtained in initial calibration step, some particles can already be reliably tracked. At this step, we assume that some existing trajectories (longer than 50 frames in this study) were successfully measured, and that the resolved trajectories contained true 3D positions in lab-space. The updated calibration is done by minimizing the calibration error using these 3D positions together with the corresponding 2D positions in each camera. The output of this step is the updated camera models for both cameras including the nonlinear correction term $\overrightarrow{e}\left(\eta , \zeta \right)$ with a calibration error of less than 1 pixel.

It is important to note that steps 3, 4, and 5 follow an iterative process. We proceed to step 6 only when the number of trajectories and the average temporal trajectory length reach a certain level after step 4. For example, in the experiment shown in Fig. 4a–c, the levels are set as 40,000 total trajectories and an averaged temporal trajectory length of 10 frames.

6. Trajectory smoothing

Based on the trajectories from step 4, linear fitting is implemented to calculate the particle velocity. Trajectories shorter than a threshold (typically 10–30 frames) are discarded. In the experiment shown in Fig. 4a–c, the threshold is set as 20 frames. For more complex particle trajectories, MyPTV toolbox also supports polynomial fitting for calculating particle velocity and acceleration.

## Appendix C: Individual behavior of tracked particles

Based on the 4D trajectories shown in Fig. 3 (main text), we can analyze the trajectories in more detail both vertically and radially. Figure 10 shows the relationship between the vertical position and the time, as well as the radial position and the vertical position, respectively, where the colors of the curves are consistent with the corresponding particles shown in Fig. 3c and f (main text). Regarding three tracked SHGS particles in glycerol, Fig. 10a gives both detected particle positions (markers) and the positions in linear fitted trajectories (solid line), where linearity can be clearly observed. Figure 10b shows that the vertical velocities and the radial positions of these three particles are independent of their vertical positions. Regarding three tracked SHGS particles in the blood, they also keep a constant vertical velocity, as linearity can also be clearly observed in Fig. 10c. Figure 10d shows that particle 1 moves toward the center with Δ*r*/Δ*z* = -− 0.064, while particle 3 moves toward the wall with Δ*r*/Δ*z* = 0.018, which indicates a migration behavior.

## Appendix D: Error analysis of tracked particles

To calculate the uncertainty of the position of a tracked particle, the coordination systems, viewed from the direction perpendicular to the optical plane, are defined as shown in Fig. 11a. The red and the green arrow denote the direction of two X-ray beamlets generated by our XMPI setup, respectively. Coordinates (x,y,z), $({x}_{1}, {y}_{1}, {z}_{1})$ and $({x}_{2}, {y}_{2}, {z}_{2})$ denote lab coordinate, camera 1 coordinate and camera 2 coordinate, respectively. ${\varphi}_{1}$ and ${\varphi}_{2}$ denote the angle between ${x}_{1}$ and x direction, ${x}_{2}$ and x direction, respectively. $\phi ={\varphi}_{2}-{\varphi}_{1}$ denotes the angular separation of two projections in the XMPI setup, which is 47.7 degrees in the experiments conducted in this work. Note that the cameras can only provide 2D images, so ${x}_{1}$ and ${x}_{2}$ coordinates cannot be directly read from the acquired images.

Suppose we have a particle located at r=(x,y) in XY plane. From two cameras, ${y}_{1}$ and ${y}_{2}$ can be read to determine the particle position, as it is obvious from Fig. 11a that ${y}_{1}=R-{d}_{1}$ and.${y}_{2}=R-{d}_{2}$. Describing the particle position in two camera coordinates $\left({x}_{1},{y}_{1}\right)$ and$\left({x}_{2},{y}_{2}\right)$, respectively, we have:

3 $$\left\{\begin{array}{c}x={x}_{1}\mathrm{cos}{\varphi}_{1}-{y}_{1}\mathrm{sin}{\varphi}_{1}={x}_{2}\mathrm{cos}{\varphi}_{2}-{y}_{2}\mathrm{sin}{\varphi}_{2}\\ y={x}_{1}\mathrm{sin}{\varphi}_{1}+{y}_{1}\mathrm{cos}{\varphi}_{1}={x}_{2}\mathrm{sin}{\varphi}_{2}+{y}_{2}\mathrm{cos}{\varphi}_{2}\end{array}\right.$$

After eliminating ${x}_{1}$ and ${x}_{2}$, which cannot be directly read from the images, we have:

4 $$\left\{ {\begin{array}{*{20}l} {x = \frac{{y_{1} \sin \varphi _{1} + y_{1} \sin \left( {\varphi _{1} - 2\varphi _{2} } \right) + y_{2} \sin \varphi _{2} - y_{2} \sin \left( {2\varphi _{1} - \varphi _{2} } \right)}}{{\cos \left( {2\varphi _{1} - 2\varphi _{2} } \right) - 1}}} \\ {y = \frac{{2\left( {y_{1} \sin \varphi _{2} - y_{2} \sin \varphi _{1} } \right)\sin \left( {\varphi _{1} - \varphi _{2} } \right)}}{{\cos \left( {2\varphi _{1} - 2\varphi _{2} } \right) - 1}}} \\ \end{array} } \right.$$

In Eq. (4), the uncertainty of both x and y originate from the uncertainty of ${y}_{1}$ and ${y}_{2}$. Therefore, we have:

5 $$\left\{\begin{array}{*{20}l}{\sigma}_{x}=\sqrt{{\left(\frac{\partial x}{\partial {y}_{1}}\cdot {\sigma}_{{y}_{1}}\right)}^{2}+{\left(\frac{\partial x}{\partial {y}_{2}}\cdot {\sigma}_{{y}_{2}}\right)}^{2}}\\ {\sigma}_{y}=\sqrt{{\left(\frac{\partial y}{\partial {y}_{1}}\cdot {\sigma}_{{y}_{1}}\right)}^{2}+{\left(\frac{\partial y}{\partial {y}_{2}}\cdot {\sigma}_{{y}_{2}}\right)}^{2}}\end{array}\right.$$

Furthermore, for calculating the uncertainty of the radial position in XY plane, we have:

6 $$\left\{\begin{array}{*{20}l}r=\sqrt{{x}^{2}+{y}^{2}}\\ {\sigma}_{r}=\frac{\sqrt{{\sigma}_{x}^{2}{x}^{2}+{\sigma}_{y}^{2}{y}^{2}}}{r}\end{array}\right.$$

In PTV conducted in this work, we focus on getting the radial position of each particle, regardless of the orientation of the lab coordinate in xy plane (defined by ${\varphi}_{1}$). Therefore, for simplicity in estimating ${\sigma}_{r}$, we select ${\varphi}_{1}=(90^\circ -\phi )/2$ so that ${\sigma}_{x}={\sigma}_{y}={\sigma}_{r}/\sqrt{2}$

Given that both cameras are identical and independent regarding their detection efficiency and spatiotemporal resolution despite their slightly different sample-detector distances, the uncertainties of ${y}_{1} \text{ and } {y}_{2}$ are the same. Given that the cameras have the same resolution in both axes, we further assume that such uncertainty is the same as ${\sigma}_{z}$, which describes the uncertainty of vertical position in the lab coordinate. We estimate ${\sigma}_{z}$ by considering three main sources of error, as discussed in Sect. 3.1.2. The first is related to the instability of the beamlet, giving ${\sigma}_{1}=1 \text{ pix}$. The second is related to the particle segmentation as described in step 2 of the MyPTV analysis. Considering the difficulty of deciding the center of each particle when several particles contact or interact with each other, a reasonable uncertainty of ${\sigma}_{2}=2 \text{ pix}$ is used. The third comes from the camera calibration process, resulting in ${\sigma}_{3}=1 \text{ pix}$. Therefore, we have:

7 $${\sigma}_{{y}_{1}}={\sigma}_{{y}_{2}}={\sigma}_{z}=\sqrt{{\sigma}_{1}^{2}+{\sigma}_{2}^{2}+{\sigma}_{3}^{2}}=2.45 \text{ pix}$$

Using Eqs. (3–6), the relationship of ϕ and the ratio ${\sigma}_{r}/{\sigma}_{z}$ is shown in Fig. 11b. The uncertainty in radial position decreases when angular separation ϕ increases toward $90^\circ$. In this work,$\phi =47.7^\circ$, so the uncertainties of the position of the tracked particle are: ${\sigma}_{z}=2.45 \text{ pix};$

$${{\sigma}_{r}=\sqrt{2}\sigma }_{x}={\sqrt{2}\sigma }_{y}=1.91 {\sigma}_{z}=4.7 \text{ pix}.$$

According to step 6 of the MyPTV analysis, linear fitting of the trajectory is implemented to calculate the velocity. Suppose we have multiple points $\left({z}_{i},{t}_{i}\right)$ with i=1,2,⋯,n for a certain trajectory, where n indicates the total number of time points per trajectory; ${z}_{i}$ is the vertical position given by the tracking results at time ${t=t}_{i}$. The vertical velocity ${u}_{z}$ is the slope of the fitted line with an uncertainty ${\sigma}_{{u}_{z}}$ given in Eq. (8), which is dependent on both ${\sigma}_{z}$ and the temporal length of the trajectory (the number of frames per trajectory) as is shown in Fig. 11c. In the experiment presented in Fig. 4a–c, the average Pearson correlation coefficient for all 2471 tracked particles is 0.9992, indicating that the acceleration of particles in z direction is negligible.

8 $$\sigma_{{u_{z} }} = \sigma_{z} \cdot \sqrt {\frac{1}{{\sum \left( {t_{i} - \overline{t}} \right)^{2} }}}$$

To avoid underestimating the error in calculating the vertical velocity, we use the trajectory with minimal temporal length to estimate ${\sigma}_{{u}_{z}}$ for all the tracked particles in each experiment. For example, the minimal trajectory length is 20 frames in the experiment presented in Fig. 4a–c, resulting in ${\sigma}_{{u}_{z}}=0.005\,\text{ mm/s}$.

To sum up, in this work, we can estimate that the vertical position uncertainty ${\sigma}_{z}=2.45 \text{ pix}=3.2 \text{ } \upmu \mathrm{m}$; the radial position uncertainty is ${\sigma}_{r}=4.7 \text{ pix}=6.1 \text{ } \upmu \mathrm{m}$. The uncertainty of vertical velocity depends on the temporal length of the trajectory. A longer trajectory results in lower uncertainty.

## List of symbols

*g*: Gravitational acceleration (m/s^2^)
*R*_p_: Mean radius of particles (m)
*R*: Inner radius of Kapton tube (m)
*r*: Radial position from tube center (m)
Δ*r*: Particle-free layer thickness closest to tube wall (m)
*μ*: Viscosity (Pa·s)
*ρ*: Density of SHGS (kg/m^3^)
*ρ*_*s*_: Density of glycerol (kg/m^3^)
*ρ*_N_: Number density of particles (1/m^3^)
*Q*: Flow rate (m^3^/s)
*V*_sed_: Theoretical sedimentation velocity (m/s)
*V*_max_: Theoretical maximum velocity (m/s)
Re: Reynolds number (dimensionless)
*x, y*: Horizontal coordinates in lab-frame (m)
*z*: Vertical coordinate (m)
*u*_*z*_: Vertical velocity (m/s)
*x*_1,2_, * y*_1,2_: Horizontal coordinates in projection 1 (P1) and projection 2 (P2) (m)
*E*: Energy of the X-ray beam (J)
Δ*E*: Bandwidth of X-ray beam (J)

## Abbreviations

3D: Three dimensional (space)
4D: Four-dimensional (space + time)
CFD: Computational fluid dynamics
ECT: Electrical capacitance tomography
MRV: Magnetic resonance velocimetry
OCT: Optical coherence tomography
OF: Optical flow analysis
P1, P2: Projection 1, Projection 2
PIV: Particle image velocimetry
PTV: Particle tracking velocimetry
SHGS: Silver-coated hollow borosilicate glass spheres
UDV: Ultrasound Doppler velocimetry
XCT: Computed tomographic X-ray imaging
XFEL: X-ray free-electron laser
XMPI: X-ray multi-projection imaging
